# Elucidating the correlation between treatment with tyrosine kinase inhibitors and mean platelet volume in patients with metastatic renal cell cancer

**DOI:** 10.3892/ol.2014.2495

**Published:** 2014-09-03

**Authors:** SEYDA GUNDUZ, HASAN MUTLU, MUKREMIN UYSAL, HASAN SENOL COSKUN, HAKAN BOZCUK

**Affiliations:** 1 Department of Medical Oncology, Akdeniz University Hospital, Antalya 07070, Turkey; 2 Department of Medical Oncology, Afyon Kocatepe University Ahmet Necdet Sezer Research and Practice Hospital, Afyon 3000, Turkey

**Keywords:** tyrosine kinases inhibitors, mean platelet volume, thrombosis

## Abstract

Patients with cancer are at increased risk of thrombosis. Additionally, an increased mean platelet volume (MPV) has been demonstrated to be associated with thromboembolism. Tyrosine kinase inhibitors (TKIs) may modulate the activation of systemic coagulation in cancer patients, rendering them more susceptible to thromboembolism. The aim of the current study was to investigate the association between antiangiogenic TKIs and MPV. A total of 45 patients with metastatic renal cell carcinoma (RCC), who were treated with TKIs and were patients at the Akdeniz University Hospital and Afyon Kocatepe University Ahmet Necdet Sezer Research and Practice Hospital, were retrospectively reviewed. The results prior to treatment and after three months for the MPV values and platelet levels were evaluated. The MPV values increased following the treatment with TKIs; however, no statistically significant difference was observed between the baseline and three month values (P=0.286). Conversely, a significant decrease was observed in the platelet levels following treatment (P=0.005). Treatment with TKIs in patients with metastatic RCC caused a modest increase in MPV, which is an indicator of thrombocytic reactivity; however, further studies are required to validate these results.

## Introduction

The mean platelet volume (MPV) is a platelet volume index (1). Classically, MPV was recognized as a characteristic of platelet activation. Larger platelets are more reactive than smaller ones; therefore, their release of chemical mediators is easier in response to endogenous or exogenous stimuli (2). MPV has been observed to correlate with various thromboembolic disorders, and increased MPV associated with thromboembolism has been reported in patients with myocardial infarction, cerebrovascular thromboembolism, diabetes mellitus and smokers (3–6). In addition, the decrease of MPV has been previously reported in a cancer patient with thromboembolic events who was undergoing chemotherapy with bevacizumab (7). Recent studies have also revealed that the MPV and MPV/platelet count (PC) ratio can predict the long-term mortality in patients with advanced non-small cell lung cancer (NSCLC) (8).

Vascular endothelial growth factor (VEGF) is primary target in the antiangiogenic treatment of solid tumors (9). Clinical trials have shown that treatment with antiangiogenic agents, including sorafenib, sunitinib, bevacizumab and pazopanib, in advanced renal cell carcinoma (RCC) has exhibited consistent prolongation of the progression-free survival and, in certain cases, overall survival in treatment-naive and previously treated patients (10).

The inhibition of angiogenesis is associated with an increased risk of arterial thromboembolic events (ATE) and venous thromboembolic events (VTE) (11). VEGF receptor tyrosine kinase inhibitors (TKIs) may modulate the activation of systemic coagulation in cancer patients, rendering them more susceptible to thromboembolism (12,13). Clinical trials have reported that bevacizumab was significantly associated with an increased risk of developing VTE in patients with cancer (14). In this analysis, the incidence of all-grade and high-grade VTE was 11.9 and 6.3%, respectively. A meta-analysis to assess the risk of ATE reported that treatment with sunitinib and sorafenib is associated with a three-fold increase in the risk of ATE, with an overall incidence of 1.3% in patients with RCC (15).

The aim of the current study was to evaluate whether antiangiogenic TKIs, such as sunitinib, sorefenib and pazopanib, have an effect on MPV values in patients with metastatic RCC.

## Materials and methods

### Patients

A total of 45 patients with metastatic RCC were reviewed from the Department of Oncology, Akdeniz University Hospital (Antalya, Turkey) and the Department of Oncology, Afyon Kocatepe University Ahmet Necdet Sezer Research and Practice Hospital (Afyon, Turkey) between May 2009 and September 2013, retrospectively. All patients received interferon-α (IFN-α) therapy until progression or intolerance prior to treatment with TKIs. Following the treatment with IFN-α, antiangiogenic TKIs (sunitinib, sorafenib or pazopanib) were offered to the all patients. This study was approved by the ethics committee of Akdeniz University (Antalya, Turkey) and written informed consent was obtained from all patients.

### Treatment plan

In a treatment group of metastatic RCC patients, the MPV values prior to treatment and after three months of treatment with sunitinib, sorafenib and pazopanib were compared. Additionally, the platelet levels at the baseline and three months were compared.

### Statistical analysis

Statistical analyses were performed using SPSS software, version 20.0 (IBM, Armonk, NY, USA). The variables were investigated using visual (histograms and probability plots) and analytical (Kolmogorov-Simirnov/Shapiro-Wilk test) methods to determine whether the values were normally distributed. The data are presented as the mean ± standard deviation for normally distributed variables (MPV measurements). Paired Student’s t-test was used to compare the measurements at two time points (baseline and three months) for MPV and PC. P<0.05 was considered to indicate a statistically significant difference.

## Results

### Patient characteristics

The mean follow-up duration was 40.8 months. The median age of the patients was 63 years (range, 41–90). Of the patients, 75.6% were male and 24.4% were female; 77.8% of patients used sunitinib, 11.1% used sorafenib and 11.1% used pazopanib. The remaining characteristics of the patients prior to treatment are shown in Table I.

### MPV values at baseline and three months

**Figure 1:**
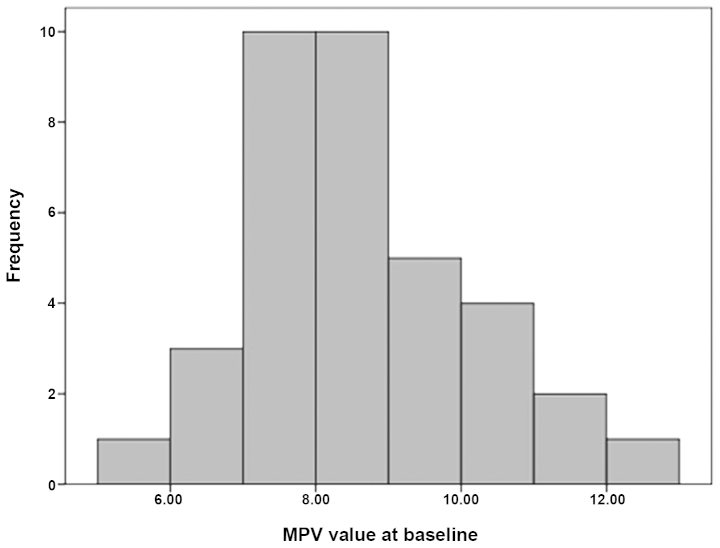
MPVs prior to treatment. MPV, mean platelet volume.

**Figure 2:**
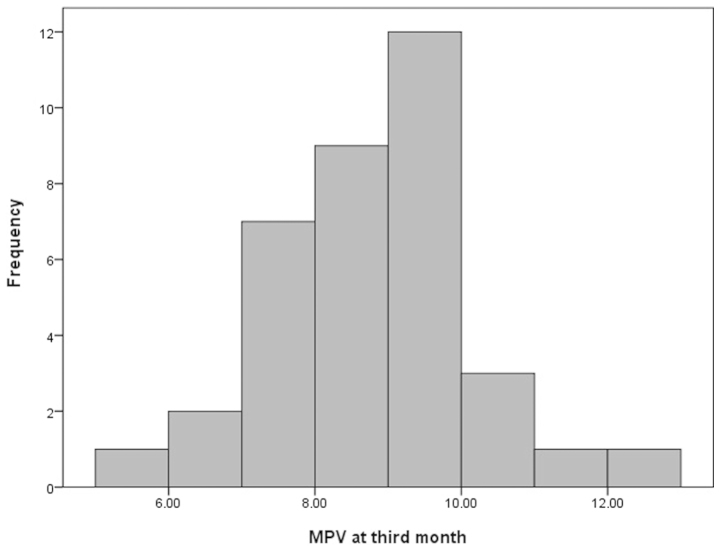
MPVs after three months of treatment with tyrosine kinase inhibitors. MPV, mean platelet volume.

MPV levels were normally distributed (P=0.372 and P=0.615, respectively) according to the Shapiro-Wilk test (n<50). Histograms for the MPV values are shown in Figs. 1 and 2. The mean MPV values at baseline and three months were 8.53±1.44 and 8.75±1.30, respectively. MPV levels increased with the treatment with TKIs; however, no statistically significant difference was identified between MPV values at the baseline and three months of the treatment (P=0.286). Conversely, a statistically significant decrease in platelet levels was observed following treatment (P=0.005). The values for PC and MPV are presented in Table II.

## Discussion

To the best of our knowledge, this study is the first study to evaluate the MPV values in patients with metastatic RCC, receiving antiangiogenic TKIs.

In the presence of increased MPV levels, wide platelets with dense granules, containing increased thromboxane A2 may be observed in the blood. The *in vitro* response to adenosine 50-diphosphate and collagen, as well as a tendency towards aggregation, are also increased (16). Several reports have indicated that an elevation of MPV is closely associated with the severity and prognosis of cerebra-and cardiovascular disorders (16,17). Osada *et al* (18) showed that the MPV was higher in patients with gastric cancer than in control patients. Another two trials demonstrated that the MPV and MPV/PC ratio were elevated in patients with hepatocellular carcinoma and NSCLC (7,19). By contrast, a study by Mutlu *et al* (20) analyzed the MPV in patients with metastatic colon cancer who were treated with bevacizumab. A decrease in PC and MPV was identified during the treatment period (8). Recently Braekkan *et al* (21) investigated MPV as a potential risk factor for VTE. The results demonstrated that patients with an MPV of >9.5 had a significantly (1.5-fold) increased risk of VTE, compared with an MPV of <8.5. Antiplatelet drugs reduce the risk of arterial cardiovascular events and VTE (21). MPV levels have been shown to be decreased in patients with cancer in clinical trials (8,20). In the current study, the MPV exhibited a tendency to be increased in patients with metastatic RCC.

Bevacizumab is an antiangiogenic agent that has exhibited activity as a cancer treatment; however, significant adverse events, including hemorrhage and thrombosis, have also been observed during treatment. A previous study demonstrated a decrease in MPV levels in cancer patients who use chemotherapy regimens with bevacizumab (7). The evidence for the use of aspirin prophylaxis for ATE for patients using bevacizumab is conflicting. Scappatici *et al* (22) reported marginally more grade 3 and 4 bleeding events among aspirin users on bevacizumab than in the control subjects (3.7 vs. 1.8%). Conversely, a pooled analysis of low-dose aspirin for primary prophylaxis for ATEs in patients undergoing chemotherapy with bevacizumab did not identify any increased bleeding risk (23). Tebbutt *et al* (24) demonstrated that the rate of ATE was moderately higher in patients on aspirin in combination with bevacizumab. A clinical study demonstrated a decrease in MPV during the treatment period with bevacizumab (7). In the current study, the MPV value was further increased in patients with metastatic RCC. This result may be due to the different mechanisms of action of bevacizumab and antiangiogenic TKIs.

According to the results of this study, MPV levels were increased by the treatment with TKIs after three months; however, the difference was not statistically significant. Further studies are required to validate the use of TKIs to increase the MPV values, which act as indicators of thrombocytic reactivity. We hypothesize that the use of aspirin for thromboprophaxis may be of additional benefit to these patients.

## Figures and Tables

**Table I:** Baseline characteristics of patients.

	Patients	
	
Characteristics	n=45	%
Gender, n		
Female	11	24.4
Male	34	75.6
Age, years		
Median	63	
Range	41–90	
Metastatic site, n		
Visceral	24	53.3
Bone	8	17.8
Bone and visceral	8	17.8
Missing	5	11.1
ECOG performance status, n		
0	5	13.2
1	14	36.8
2	11	28.9
3	8	21.1
Tyrosine kinase inhibitors, n		
Sunitinib	35	77.8
Sorafenib	5	11.1
Pazopanib	5	11.1

**Table II:** Levels of mean platelet volume and platelet count.

Parameters	Prior to treatment, mean	Three months, mean	P-value
Platelet count, 10^3^/ml	281.527±111.460	220.000±128.751	0.005
Female	293.750±91.610	223.875±201.447	0.167
Male	278.035±117.770	218.892±104.698	0.019
Mean platelet volume, fl	8.52±1.43	8.75±1.30	0.286
Female	8.30±1.80	9.15±1.11	0.917
Male	8.57±1.34	8.63±1.34	0.738
